# Cardiac magnetic resonance imaging for discrimination of hypertensive heart disease and hypertrophic cardiomyopathy: a systematic review and meta-analysis

**DOI:** 10.3389/fcvm.2024.1421013

**Published:** 2024-08-02

**Authors:** Qingyuan Zhao, Zhiyu Chen, Chengcheng Qi, Sunan Xu, Ruichen Ren, Wenting Li, Xiaoxue Zhang, Yang Zhang

**Affiliations:** Department of Radiology, Qilu Hospital of Shandong University, Jinan, China

**Keywords:** hypertensive heart disease, hypertrophic cardiomyopathy, cardiac magnetic resonance imaging, T1 mapping, ECV, strain

## Abstract

**Introduction:**

Differentiating hypertensive heart disease (HHD) from hypertrophic cardiomyopathy (HCM) is crucial yet challenging due to overlapping clinical and morphological features. Recent studies have explored the use of various cardiac magnetic resonance (CMR) parameters to distinguish between these conditions, but findings have remained inconclusive. This study aims to identify which CMR parameters effectively discriminate between HHD and HCM and to investigate their underlying pathophysiological mechanisms through a meta-analysis.

**Methods:**

The researchers conducted a systematic and comprehensive search for all studies that used CMR to discriminate between HHD and HCM and calculated the Hedges'g effect size for each of the included studies, which were then pooled using a random-effects model and tested for the effects of potential influencing variables through subgroup and regression analyses.

**Results:**

In this review, 26 studies encompassing 1,349 HHD and 1,581 HCM cases were included for meta-analysis. Analysis revealed that HHD showed a significant lower in T1 mapping (g = −0.469, *P* < 0.001), extracellular volume (g = −0.417, *P* = 0.024), left ventricular mass index (g = −0.437, *P* < 0.001), and maximal left ventricular wall thickness (g = −2.076, *P* < 0.001), alongside a significant higher in end-systolic volume index (g = 0.993, *P* < 0.001) and end-diastolic volume index (g = 0.553, *P* < 0.001), compared to HCM.

**Conclusion:**

This study clearly demonstrates that CMR parameters can effectively differentiate between HHD and HCM. HHD is characterized by significantly lower diffuse interstitial fibrosis and myocardial hypertrophy, along with better-preserved diastolic function but lower systolic function, compared to HCM. The findings highlight the need for standardized CMR protocols, considering the significant influence of MRI machine vendors, post-processing software, and study regions on diagnostic parameters. These insights are crucial for improving diagnostic accuracy and optimizing treatment strategies for patients with HHD and HCM.

**Systematic Review Registration:**

https://www.crd.york.ac.uk/prospero/display_record.php?ID=CRD42023470557, PROSPERO (CRD42023470557).

## 1 Introduction

Hypertensive heart disease (HHD) and hypertrophic cardiomyopathy (HCM) are both characterized by left ventricular hypertrophy (LVH) but have distinct pathogenesis and clinical management strategies. HHD results from prolonged hypertension leading to left ventricular remodeling, and its treatment focuses on controlling blood pressure for a generally better prognosis (1). In contrast, HCM is an inherited disorder caused by mutations in myocardial sarcomere genes, requiring management of symptoms and prevention of sudden cardiac death, often involving invasive interventions, leading to a variable prognosis depending on disease severity and risk factors (2). However, distinguishing between HHD and HCM remains a significant clinical challenge, particularly because overlapping LVH and multiple forms of HCM (3), often resulting in diagnostic ambiguity when relying on a single morphological index. This challenge is compounded by the high prevalence of hypertension in HCM patients (4), frequent absence of family history in HCM (5), and limitations in genetic testing (6). These challenges underscore the need for precise diagnostic tools and comprehensive clinical evaluation to ensure optimal patient outcomes.

Cardiovascular Magnetic Resonance (CMR) has emerged as a pivotal tool in the diagnosis of cardiomyopathies, providing detailed insights into the etiology and pathophysiology of these conditions (7). Current studies increasingly explore HHD and HCM using CMR, focusing on (1) extent of diffuse fibrosis (8, 9), native T1mapping and myocardial extracellular volume (ECV); (2) myocardial deformation damage: global radial strain (GRS), global circumferential strain (GCS), and global longitudinal strain (GLS); (3) hypertrophy patterns and systolic-diastolic disorders: left ventricular mass index (LVMI), maximal left ventricular wall thickness (maximal LVWT), and end-diastolic and end-systolic volume index (ESVI, EDVI). Despite these advancements, there is no consensus on the utility of these CMR parameters in reliably distinguishing HHD from HCM or their ability to reflect distinct pathophysiological characteristics.

This study is the first to perform a quantitative meta-analysis of the utility of CMR in discriminating HHD from HCM and to explore potential influencing factors using subgroup and regression analyses. This study aims to (1) evaluate CMR’s effectiveness in differentiating HHD from HCM for clinical application, and (2) investigate their potential pathophysiological differences.

## 2 Methods

### 2.1 Search strategy and study selection

The protocol of this meta-analysis is available at PROSPERO (CRD42023470557). Relevant literature was methodically retrieved from PubMed, Embase, Web of Science, and Cochrane Library up to September 2023, following the PRISMA guidelines (10). To avoid erroneous omissions, the search strategy encompassed three core themes of this article: HHD, HCM, and CMR (see Supplementary Table S1). Inclusion criteria for studies were: (1) inclusion of human subjects with clearly defined HHD and HCM criteria; (2) comparison of HHD and HCM; (3) provision of specific quantitative MRI parameters (T1mapping, ECV, GRS, GCS, GLS, LVMI, Maximal LVWT, ESVI, EDVI); (4) clear description of MRI techniques and protocols used for parameter measurements; and (5) publication as a peer-reviewed article in English. Exclusion criteria included: (1) reviews, guidelines, conference proceedings, animal experiments, or case reports; (2) absence of HHD or HCM data; (3) MRI parameters outside the research scope; (4) incomplete or unclear methodology regarding MRI parameter acquisition; and (5) overlapping data from the same research group without clear distinction or unique data. Two reviewers (Q.Z. and Z.C.) independently conducted literature searches and study selection.

### 2.2 Quality assessment

Risk of bias and concerns over applicability of the included studies were assessed by two reviewers separately using the Joanna Briggs Institute Critical Appraisal tools (JBI) (11), which evaluates the credibility, relevance, and results of studies via eight questions, detailed in Supplementary Table S2. The visual inspection method for funnel plot symmetry (12) and egger's test (13) were used to assess the potential publication bias. For analyses where publication bias existed (two-tailed *p* < 0.05), we used Duval and Tweedie's “trim and fill” method to adjust the impact of publication bias (14).

### 2.3 Data extraction

Data from eligible studies were extracted by one author (Q.Z.) and double-checked by a second (Z.C.). Following data were extracted from the included studies: (1) study characteristics: author, year, institution, study design (retrospective or prospective); (2) patient characteristics: inclusion/exclusion criteria, subgroups, sample sizes, age, gender, ejection fraction (EF), and complications (diabetes, dyslipidemia, smoking); (3) parameters: T1 mapping, ECV, GRS, GCS, GLS, LVMI, Maximal LVWT, ESVI, EDVI; (4) MR features: field strength, vendor, scanner, scan sequence; (5) post-processing features: software, method. Studies subdividing HCM into subgroups (15, 16), were consolidated into one group according to Supplementary Methods A. For studies reporting median, minimum and maximum rather than mean and SD (17, 18, 19), mean, and if necessary SD, were calculated according to Supplementary Methods B. For a study reporting mean and standard error of the mean (SEM) (20), conversion was performed according to Supplementary Methods C. The multicenter study (21) provided independent T1 mapping values for HHD and HCM patients at different centers and different field strengths, and was therefore considered as multiple studies.

### 2.4 Data analysis

All statistical analyses were conducted using Comprehensive Meta Analysis (Version 3.3) software. For each parameter, Hedges'g values within 95% confidence intervals (CI) were calculated for the included studies and then were pooled through a random-effects model to account for between-study heterogeneity. Compared to the fixed effects model, the random effects model is more conservative, yields wider CIs for the pooled effects, and allows conclusions to be generalized to a wider range of situations (22). Hedges'g was preferred over Cohen's d for computing standardized mean difference (SMD), as the latter may inaccurately estimate effect sizes in studies with small sample sizes (23). Hedges'g values of 0.2, 0.5, and 0.8 correspond to small, medium, and large effect sizes (24).

The significance of heterogeneity was obtained by Q test, *τ*^2^ and *I*^2^ values, which measure the true heterogeneity resulting from between-study variance rather than sampling error or chance. *I*^2^ values of 25%, 50% and 75% indicate low, medium and high proportions of heterogeneity (25), respectively. High heterogeneity indicates substantial variability among included studies, potentially undermining the analysis's conclusion reliability. Variability may stem from diverse factors, including different study designs, patient characteristics and technical approaches. Subgroup and regression analyses were used to explore potential influencing factors and to find sources of heterogeneity. The covariates are considered influencing factors if they lead to inconsistency with the overall effect. If no significant intragroup heterogeneity (*P* > 0.05) emerged in all subgroups, the covariate used for grouping were deemed a source of heterogeneity. Subgroup-analyses were conducted for MR field strength (1.5 T or 3 T), vendors of MR (Philips or Siemens or GE), software used for post-processing (Cvi42 or notCvi42), slices for measuring T1mapping (=1 or >1), region where the institute is located (Asia or Western), and difference in EF between HHD and HCM (*Δ*EF ≤ 5 or *Δ*EF > 5). Regression-analyses were conducted for mean age of the participants, percentage of males and mean EF of participants. A random-effects model is used to combine studies within each subgroup for subgroup-analysis and regression-analyses were performed with the Knapp-Hartung adjustment (26). Sensitivity analysis using the one-study-removed method to evaluate the impact of individual studies on the overall effect to assess whether the results were stable.

## 3 Results

### 3.1 Search results and selection

**Figure 1:**
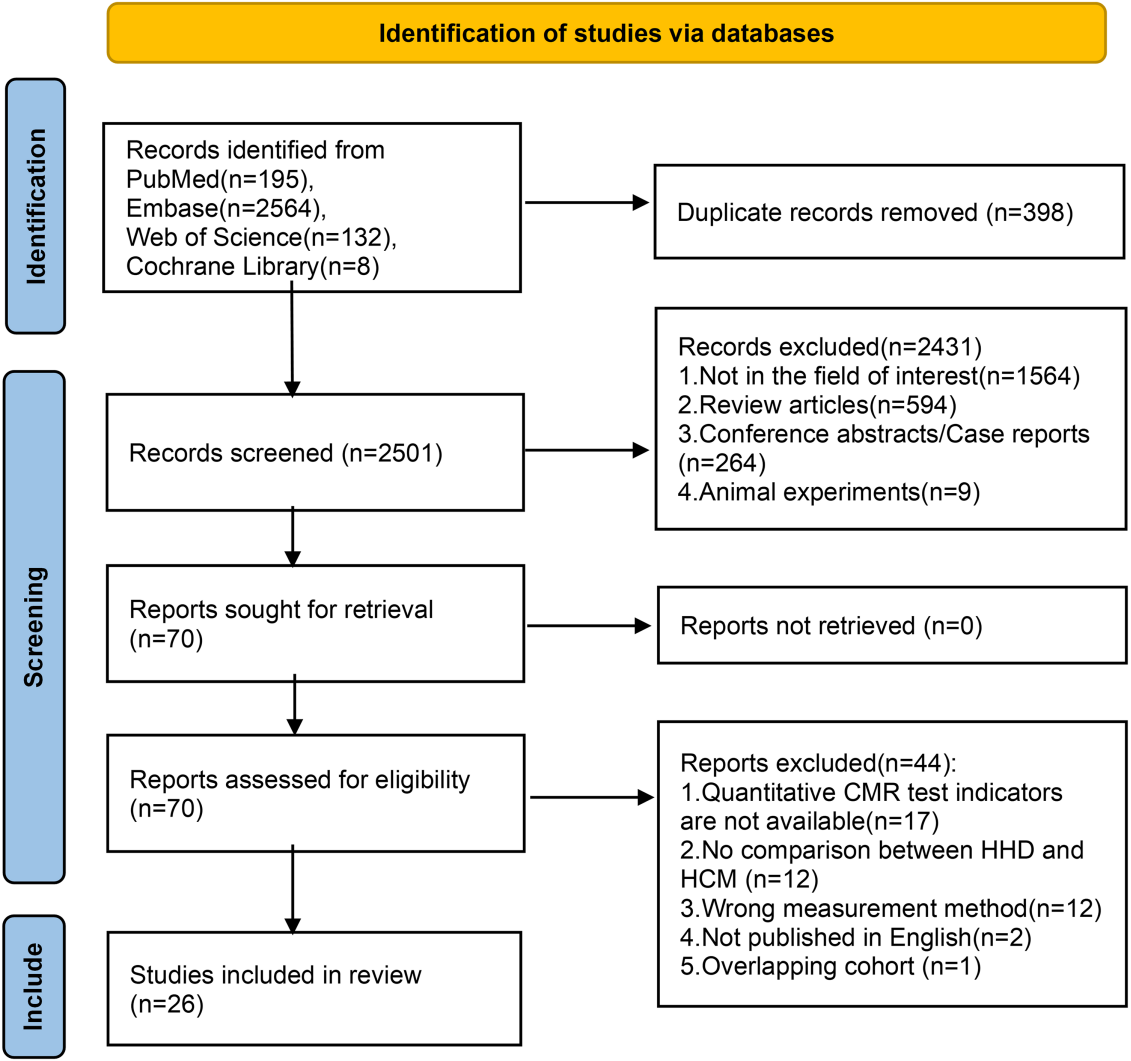
PRISMA flow diagram. HHD, hypertensive heart disease; HCM, hypertrophic cardiomyopathy.

The specific retrieval process and results are depicted in the workflow diagram (Figure 1). Initially, 2,899 articles were retrieved, including 398 duplicates. Screening of titles and abstracts led to the exclusion of 2,431 articles, yielding 70 articles for full-text review. By performing an in-depth assessment, 17 articles were excluded for not mentioning quantitative CMR data, 12 for not comparing HHD and HCM, 12 due to incorrect measurements, 2 for not publishing in English, 1 for duplicate cohorts (27), leaving 26 articles for quantitative meta-analysis. There was almost perfect agreement (Cohen's kappa = 0.878) (28) between the two reviewers, and any discrepancies [notably a study (29) disputed in the study inclusion session] have been resolved by discussion or consulting a third senior investigator (Y.Z.).

### 3.2 Characteristics of the included studies

The characteristics and demographics of the 26 included studies are detailed in Table 1, while images acquisition and post-processing details are in Table 2. These studies collectively involved 2,930 patients, comprising 1,349 with HHD and 1,581 with HCM. The mean age ranges were 45–66.2 years for HHD and 39.4–64.4 years for HCM. Excluding one study (40) that did not report the male proportion in the HHD group, the HHD group comprised 69.8% males, and the HCM group 65.3% males. The mean EF, reported in all but three studies (40, 46, 44), ranged from 28.2%–76% for HHD and 44.2%–76.1% for HCM. Excluding one study (33) that did not report the MR field strength, 14 studies used 3 T, 9 studies used 1.5 T, and 2 studies used both 1.5 T and 3 T. Most studies employed the MOLLI sequence for T1 mapping, but one study (38) used the STONE sequence. For measuring T1mapping, most studies selected 3 LV short-axis slices (basal, mid, and apex), but four studies (21, 33, 42, 36), selected a single mid-ventricular slice, and one study (38) selected 5 slices.

**Table 1:** The study characteristics and demographic characteristics of patients with HHD and HCM in 26 studies.

Study	Study design	Sample size (males)		Age (year)	EF (%)	Dyslipi-demia	Diabetes	Smoking	Parameters assessed by study
		HHD	HCM						
Kong et al. (16)	Retrospective	33 (23)	75 (49)^b^	56/55.7^b^	65/75.5^b^	12/13^b^	5/8^b^	4/16^b^	T1mapping, GRS, LVMI, Maximal LVWT, ESVI, EDVI
Hao et al. (29)	Retrospective	31 (16)	33 (18)	46.6/44.9	55.5/55	3/3	5/5	2/3	GRS, GCS, GLS
Wang et al. (20)	Retrospective	59 (40)	128 (75)	45/50	51/66	13/16	14/18	NA	T1mapping, Maximal LVWT
Yao et al. (15)	Prospective	46 (39)	68 (42)^b^	48/52.4^b^	60.1/66.3^b^	NA	9/8^b^	NA	LVMI, ESVI, EDVI
Liu et al. (30)	Retrospective	72 (56)	158 (109)	50.9/51.6	54.8/67.5	NA	NA	NA	LVMI, EDVI
Lavall et al. (31)	Retrospective	80 (69)	21 (16)	66/54	66/71	NA	2/0	NA	T1mapping, ECV, LVMI, ESVI, EDVI
Liu et al. (18)	Retrospective	45 (37)	57 (33)	47.8/55.3	39.8/64.4	24/24	8/7	21/21	GRS, GCS, GLS, LVMI, ESVI, EDVI
Liang et al. (32)	Retrospective	35 (31)	38 (25)	48.5/52.1	57.7/68.3	NA	4/8	15/8	T1mapping, ECV
Hirschberg et al. (21)	Retrospective	21 (13)	30 (20)	52.6/55.1	62.4/66.3	3/3	3/0	7/4	T1mapping
		12 (9)	17 (11)	60/55.4	56.3/74.5	3/5	2/2	0/0	T1mapping
Giusca et al. (33)	Retrospective	228 (154)	45 (28)	66.2/56	55/56	NA	53/8	NA	T1mapping, GCS, GLS, LVMI, ESVI, EDVI
Zhan et al. (34)	Retrospective	22 (20)	28 (19)	47/42	48/64	NA	NA	NA	T1mapping
Shi et al. (35)	Retrospective	29 (23)	66 (42)	60.6/52.6	65.9/75.3	9/13	10/5	12/9	T1mapping, ECV, GRS, GCS, GLS, LVMI
Arcari et al. (36)	Prospective	163 (91)	158 (92)	54/55	61/63	66/57	41/10	55/36	T1mapping, LVMI, ESVI, EDVI
Satriano et al. (37)	Prospective	30 (16)	85 (48)	59.6/50	69.3/70	10/14	6/9	5/27	GLS, LVMI, ESVI, EDVI
Neisius et al. (38)	Retrospective	53 (44)	107 (75)	60/55	63/65	35/62	13/15	NA	T1mapping, GLS, LVMI, Maximal LVWT, ESVI, EDVI
Jiang et al. (17)	Prospective	44 (33)	81 (48)	54.4/55.4	66.1^a^/76.1^a^	14/16	6/8	7/15	T1mapping, ECV, LVMI, Maximal LVWT, ESVI, EDVI
Chacko et al. (19)	Retrospective	21 (18)	41 (34)	57^a^/49^a^	62^a^/64^a^	NA	NA	NA	LVMI, Maximal LVWT, ESVI, EDVI
Arenja et al. (39)	Retrospective	38 (33)	40 (42)	57.3/56.4	57/61.8	19/21	4/6	23/14	LVMI, ESVI, EDVI
Wu et al. (40)	Prospective	20(NA)	56 (46)	55/54	NA	NA	NA	NA	T1mapping, ECV, GCS
Rodrigues et al. (41)	Retrospective	27 (20)	29 (17)	57/62	70/72	NA	NA	NA	GLS, LVMI, ESVI, EDVI
Hinojar et al. (42)	Retrospective	69 (45)	95 (64)	54/55	62/64	NA	NA	NA	T1mapping, ECV, LVMI, Maximal LVWT, EDVI
Takeda et al. (43)	Retrospective	9 (8)	11 (10)	58.9/64.4	28.2/44.2	NA	NA	NA	LVMI, ESVI, EDVI
Sipola et al. (44)	Prospective	95 (68)	24 (11)	46.3/39.4	NA	NA	NA	NA	LVMI, Maximal LVWT, EDVI
Puntmann et al. (45)	Retrospective	39 (14)	43 (25)	55/53	54/68	NA	NA	NA	GRS, GLS, LVMI, Maximal LVWT
Piella et al. (46)	Retrospective	10 (6)	12 (8)	64.1/61.4	NA	NA	NA	NA	GRS, GCS, LVMI, Maximal LVWT
Petersen et al. (47)	Retrospective	18 (15)	35 (26)	52/43	76/76	NA	NA	NA	LVMI, Maximal LVWT, ESVI, EDVI

ECV, extracellular volume; GRS, global radial strain; GCS, global circumferential strain; GLS, global longitudinal strain; LVMI, left ventricular mass index; LVWT, left ventricular wall thickness; ESVI, end-systolic-volume index; EDVI, end-diastolic-volume index; EF, ejection fraction; HHD, hypertensive heart disease; HCM, hypertrophic cardiomyopathy; NA, not applicable.

/Mean of data in HHD/HCM (Age, EF), Number of patients with specific complications in HHD/HCM (Dyslipidemia, Diabetes, Smoking).

^a^ Median.

^b^ Subgroup data combined.

**Table 2:** Image acquisition and post-processing details in the 26 studies.

Study	Institution	Magnetic resonance				Post-processing	
		B0(T)	Vendor	Scanner	Sequence	Software	Slice
Kong et al. (16)	Renji Hospital	3	Philips	Ingenia	bSSFP, MOLLI	CVI42, TomTecImagin, ViewForum	3^b^
Hao et al. (29)	Shanxi Cardiovascular Hospital	1.5	GE	Signa HDxt	SSFSE, FIESTA	CVI42	NA
Wang et al. (20)	Renji Hospital	3	Philips	Ingenia	bSSFP, MOLLI	CVI42	3^b^
Yao et al. (15)	Suzhou TCM Hospital	3	Philips	Ingenia	bSSFP	CVI42	NA
Liu et al. (30)	RenJi Hospital	3	Philips	Ingenia	bSSFP	CVI42	NA
Lavall et al. (31)	University Hospital Leipzig	3	Philips	Achieva	bSSFP, MOLLI	Intellispace	3^b^
Liu et al. (18)	The Second Affiliated Hospital of Harbin Medical University	3	Philips	Ingenia CX	bSSFP	CVI42	NA
Liang et al. (32)	Beijing Chaoyang Hospital	3	Siemens	Prisma	bSSFP, MOLLI	Syngo.via	3^b^
Hirschberg et al. (21)	University Hospital Heidelberg	1.5, 3^e^	Philips	Achieva, Ingenia	bSSFP, MOLLI	CVI42	1^a^
	McGill University Health Centre	3	Siemens	Magnetom Skyra	bSSFP, MOLLI	CVI42	1^a^
Giusca et al. (33)	GRN Hospital Weinheim	NA	NA	NA	bSSFP, MOLLI, fast-SENC	CVI42, MyoStrain	1^a^
Zhan et al. (34)	Tongji Hospital	3	Siemens	Skyra	True-FISP, MOLLI	CVI42	3^b^
Shi et al. (35)	Ren Ji Hospital	3	Philips	Ingenia	bSSFP, MOLLI	CVI42	3^b^
Arcari et al. (36)	Goethe University Hospital Frankfurt	3	Siemens	Skyra	MOLLI	NA	1^a^
Satriano et al. (37)	Stephenson Cardiac Imaging Center	3	Siemens	Prisma, Skyra	bSSFP	CVI42	NA
Neisius et al. (38)	Beth Israel Deaconess Medical Center	1.5	Philips	Achieva	bSSFP, STONE	MedIACare, CVI42, ViewForm	5^d^
Jiang et al. (17)	Renji Hospital	3	Philips	Ingenia	bSSFP, MOLLI	CVI42, ViewForum	3^b^
Chacko et al. (19)	St. Michael's Hospital	1.5	Philips	Intera	bSSFP	CVI42	NA
Arenja et al. (39)	University of Heidelberg	1.5	Philips	Achieva	bSSFP	NA	NA
Wu et al. (40)	Renji Hospital	3	Philips	Ingenia	bSSFP, MOLLI	CVI42	2^c^
Rodrigues et al. (41)	University Hospitals Bristol NHS Foundation Trust	1.5	Siemens	Avanto	bSSFP	CVI42	NA
Hinojar et al. (42)	Department of Cardiovascular Imaging, King's College London	3	Philips	Achieva	bSSFP, MOLLI	CVI42	1^a^
Takeda et al. (2013) (43)	Nippon Medical School	1.5,3^f^	Philips	NA	bSSFP	ViewForum	NA
Sipola et al. (2011) (44)	Kuopio University Hospital	1.5	Siemens	Magnetom Vision	FLASH	Numaris	NA
Puntmann et al. (45)	German Heart Institute	1.5	Philips	Achieva	bSSFP	ViewForum	NA
Piella et al. (46)	Universitat Pompeu Fabra	1.5	GE	Signa CVi-HDx	NA	NA	NA
Petersen et al. (47)	University of Oxford Centre	1.5	Siemens	Sonata	bSSFP	Argus and Syngo 2002B	NA

GE, general electric; CVI42, circle cardiovascular imaging 42; NA, not applicable.

^a^ Global T1mapping values were calculated as the single, mid-ventricular short axis slice.

^b^ Global T1mapping value were calculated from 3 short axis slices (basal, mid and apical).

^c^ Global T1mapping value were calculated from 2 short axis slices (basal and mid).

^d^ Not specify which 5 short-axis slices.

^e^ 1.5 T and 3 T MR were used for two separate patient cohorts.

^f^ 1.5 T and 3 T MR mixed in a patient cohor.

### 3.3 Main meta-analysis

**Figure 2:**
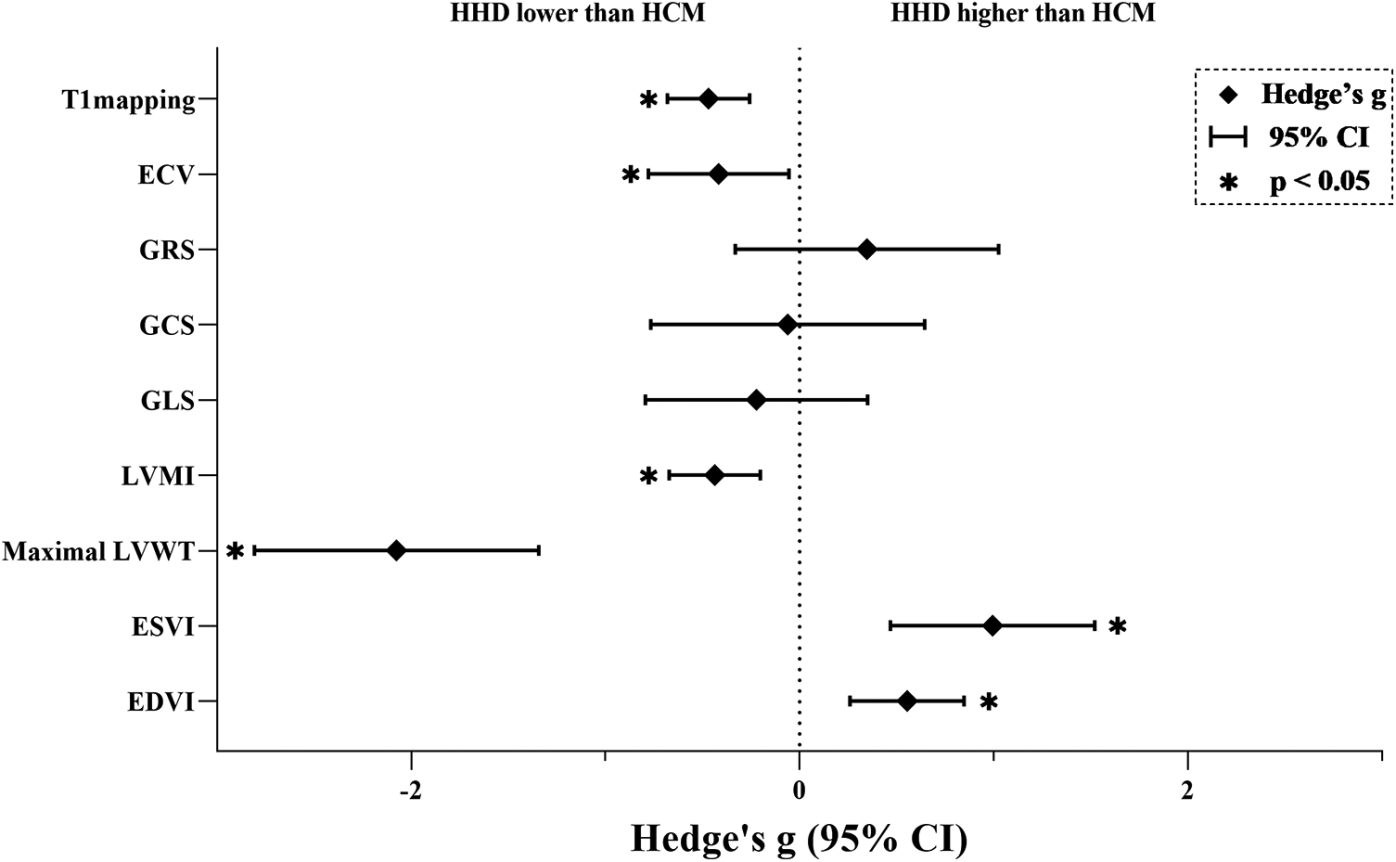
Results of the main meta-analyses. An asterisk indicates a statistically significant effect size for the parameter. ECV, extracellular volume; GRS, global radial strain; GCS, global circumferential strain; GLS, global longitudinal strain; LVMI, left ventricular mass index; LVWT, left ventricular wall thickness; ESVI, end-systolic-volume index; EDVI, end-diastolic-volume index; HHD, hypertensive heart disease; HCM, hypertrophic cardiomyopathy.

The key findings from the meta-analyses of nine CMR parameters are summarized in Figure 2; Table 3, with subgroup-analyses in the Supplementary Table S3, and regression-analysis in the Supplementary Table S4. Specific results are detailed below by grouping.

**Table 3:** Meta-analysis effect size, heterogeneity and publication bias in the 26 studies of patients with HHD and HCM.

Parameter	Number of studies reporting	Number of patients	Hedges'g			Z-value	*P*-value	Heterogeneity				Egger's test (P)
			g	Lower g	Upper g			*τ* ^2^	*I*^2^ (%)	Q	*P*	
Diffuse fibrosis												
T1mapping	15	878/945	−0.469	−0.681	−0.258	−4.352	<0.001*	0.118	74.005	53.856	<0.001	0.062
ECV	6	133/357	−0.417	−0.779	−0.055	−2.258	0.024*	0.158	78.068	22.797	<0.001	0.992
Myocardial deformation												
GRS	6	197/286	0.346	−0.331	1.024	1.001	0.317	0.644	91.863	61.448	<0.001	0.135
GCS	6	328/212	−0.061	−0.767	0.644	−0.171	0.865	0.709	92.984	71.269	<0.001	0.798
GLS	8	492/465	−0.223	−0.794	0.349	−0.763	0.445	0.634	93.570	108.863	<0.001	0.033*
Structure and function												
LVMI	20	1,161/1,269	−0.437	−0.672	−0.203	−3.654	<0.001*	0.233	85.155	127.993	<0.001	0.552
Maximal LVWT	10	510/572	−2.076	−2.808	−1.343	−5.551	<0.001*	1.311	95.392	195.315	<0.001	0.141
ESVI	14	837/871	0.993	0.467	1.519	3.699	<0.001*	0.941	95.359	280.11	<0.001	0.033*
EDVI	17	1,073/1,148	0.553	0.259	0.847	3.685	<0.001*	0.331	89.532	152.853	<0.001	0.119

ECV, extracellular volume; GRS, global radial strain; GCS, global circumferential strain; GLS, global longitudinal strain; LVMI, left ventricular mass index; LVWT, left ventricular wall thickness; ESVI, end-systolic-volume index; EDVI, end-diastolic-volume index.

/Number of patients with HHD/HCM.

* Significant at *P* < 0.05 for effect size and Egger's test.

#### 3.3.1 Analysis of diffuse fibrosis

T1mapping (N_study_ = 15, N_HHD_ = 878, N_HCM_ = 945) is significantly lower in HHD compared to HCM (g = −0.469, 95% CI: −0.681 to −0.258, *P* < 0.001). However, this effect had high heterogeneity (*I*^2^ = 74.005%, *P* < 0.001) and no evidence of publication bias (*P* = 0.062, Supplementary Figure S1A). In the T1 mapping subgroup analyses, reduced heterogeneity was observed in several subgroups: 1.5 T (*n* = 2, *I*^2^ < 0.001%), notCvi42 (*n* = 3, *I*^2^ = 34.503%), Asia (*n* = 7, *I*^2^ = 2.800%), slice >1 (*n* = 9, *I*^2^ = 33.644%), both *Δ*EF ≤ 5 (*n* = 6, *I*^2^ = 50.182%) and *Δ*EF > 5 (*n* = 8, *I*^2^ < 0.001%). Other subgroups still maintained higher levels of heterogeneity.

ECV (N_study_ = 6, N_HHD _= 133, N_HCM_ = 357) is significantly lower in HHD compared to HCM (g = −0.417, 95% CI: −0.779 to −0.055, *P* = 0.024). However, this effect had high heterogeneity (*I*^2^ = 78.068%, *P* < 0.001) and no evidence of publication bias (*P* = 0.992, Supplementary Figure S1B).

#### 3.3.2 Analysis of myocardial deformation

GRS (N_study_ = 6, N_HHD_ = 197, N_HCM_ = 286) has no significant difference in HHD compared to HCM (g = 0.346, 95% CI: −0.331 to 1.024, *P* = 0.317). This effect had high heterogeneity (*I*^2^ = 91.863%, *P* < 0.001) and no evidence of publication bias (*P* = 0.135, Supplementary Figure S1C). In the GRS subgroup analysis, heterogeneity decreased in notCvi42 (*n* = 2, *I*^2^ < 0.001%) and GE (*n* = 2, *I*^2^ < 0.001%), while remaining unchanged in other subgroups.

GCS (N_study_ = 6, N_HHD_ = 328, N_HCM_ = 212) has no significant difference in HHD compared to HCM (g = −0.061, 95% CI: −0.767 to 0.644, *P* = 0.865). This effect had high heterogeneity (*I*^2^ = 92.984%, *P* < 0.001) and no evidence of publication bias (*P* = 0.798, Supplementary Figure S1D). In the GCS subgroup analysis, a decrease in heterogeneity was noted in Western (*n* = 2, *I*^2^ < 0.001%), with other subgroups showing consistent heterogeneity.

GLS (N_study_ = 8, N_HHD_ = 492, N_HCM_ = 465) has no significant difference in HHD compared to HCM (g = −0.223, 95% CI: −0.794 to 0.349, *P* = 0.445). This effect had high heterogeneity (*I*^2^ = 93.570%, *P* < 0.001) and evidence of publication bias (*P* = 0.033, Supplementary Figure S1E). Duval and Tweedie's “trim and fill” yielded 3 potential missing studies on the left side, reducing Hedges'g to −0.705 (95% CI: −1.300 to −0.111). In the GLS subgroup analysis, both Philips (*n* = 4, *I*^2^ = 59.862%) and Siemens (*n* = 2, *I*^2^ = 68.600%) exhibited decreased heterogeneity, as did *Δ*EF > 5 (*n* = 4, *I*^2^ = 57.057%), while other subgroups maintained.

#### 3.3.3 Analysis of structure and function

LVMI (N_study_ = 20, N_HHD_ = 1,161, N_HCM_ = 1,269) is significantly lower in HHD compared to HCM (g = −0.437, 95% CI: −0.672 to −0.203, *P* < 0.001). However, this effect had high heterogeneity (*I*^2^ = 85.155%, *P* < 0.001) and no evidence of publication bias (*P* = 0.552, Supplementary Figure S1F).

Maximal LVWT (N_study_ = 10, N_HHD_ = 510, N_HCM_ = 572) is significantly lower in HHD compared to HCM (g = −2.076, 95% CI: −2.808 to −1.343, *P* < 0.001). However, this effect had high heterogeneity (*I*^2^ = 95.392%, *P* < 0.001) and no evidence of publication bias (*P* = 0.141, Supplementary Figure S1G).

ESVI (N_study_ = 14, N_HHD_ = 837, N_HCM_ = 871) is significantly higher in HHD compared to HCM (g = 0.993, 95% CI: 0.467–1.159, *P* < 0.001). However, this effect had high heterogeneity (*I*^2^ = 95.359%, *P* < 0.001) and evidence of publication bias (*P* = 0.033, Supplementary Figure S1H). Duval and Tweedie's “trim and fill” yielded 4 potential missing studies on the left-hand side, reducing Hedges'g to 0.370 (95% CI: −0.234 to 0.973).

EDVI (N_study_ = 16, N_HHD_ = 1,073, N_HCM_ = 1,148) is significantly higher in HHD compared to HCM (g = 0.553, 95% CI: 0.259–0.847, *P* < 0.001). However, this effect had high heterogeneity (I^2^ = 89.532%, *P* < 0.001) and no evidence of publication bias (*P* = 0.119, Supplementary Figure S1I). In the EDVI subgroup analysis, reduced heterogeneity was observed in Siemens (*n* = 5, *I*^2^ = 34.854%), with other subgroups showing no change.

### 3.4 Subgroup analyses and regression analysis

In the subgroup-analysis for T1mapping, results from Siemens (*n* = 4, *P* = 0.294) diverged from the overall effect. Similarly, notCvi42 (*n* = 2, *p* = 0.488) and Western (*n* = 2, *p* = 0.496) differed in ECV analyses. Philips (*n* = 4, *p* < 0.001) varied from the overall effect in GRS analyses. Western (*n* = 2, *p* < 0.001) showed a deviation in GCS analyses. The GLS analysis revealed a difference in Philips (*n* = 4, *p* = 0.020) from the overall effect. LVMI subgroup analyses indicated differences in Cvi42 (*n* = 9, *p* = 0.114), Siemens (*n* = 5, *p* = 0.465), and Asia (*n* = 7, *p* = 0.145). In ESVI and EDVI analyses, Siemens (*n* = 4, *p* = 0.940 and *n* = 5, *p* = 0.084, respectively) showed discrepancies. For all other subgroups, the results were consistent. Some of the subgroups not performed were due to the small number of studies (*n* < 2). T1 mapping, LVMI, Maximal LVWT, ESVI, and EDVI were subjected to regression-analysis revealing no significant moderating effects (all *p* > 0.05). Others were not analyzed via meta-regression due to a limited number of included studies (*n* < 10).

### 3.5 Sensitivity analyses

Sensitivity analyses, detailed in Supplementary Figure S2, showed that individual studies could impact the statistical significance in ECV. Excluding three studies (32, 40, 42), respectively led to significant changes in ECV's result. However, no individual study significantly altered the statistical overall result in other parameters.

## 4 Discussion

In this study, we conducted a comprehensive assessment of various CMR parameters to differentiate between HHD and HCM, representing the first systematic review encompassing all pertinent studies. Our findings indicated that T1mapping, ECV, LVMI, and Maximal LVWT were significantly lower in HHD than in HCM, while ESVI and EDVI were higher. In contrast, GRS, GCS, GLS showed no significant differences between the two conditions.

We further investigated various covariates potentially influencing these results through subgroup and regression analyses. Both MR machine and post-processing affect the image quality and thus the accuracy and reliability of the parameters (48), with the magnetic field strength and the vendor reflecting the former, and the post-processing software and the slices representing the latter. Moreover, differences in disease severity and basic patient characteristics also affect the final outcome, as reflected by the difference in EF between HHD and HCM and the patient's region, age, sex, and mean EF. Most analysis results aligned with the overall effect; however, discrepancies were observed in T1 mapping, GRS, GLS, LVMI, ESVI, and EDVI across different vendors, and in ECV and LVMI across software and in ECV, GCS, and LVMI across region subgroups. Vendors, post-processing software and region moderated specific parameters, which further emphasizes the multifactorial nature of variation in CMR parameters. This underscores the need for standardized CMR protocols and the importance of considering these multiple factors in clinical interpretation and future research.

Sensitivity analyses demonstrated substantial stability in parameters other than ECV. Three studies that could significantly affect the result concluded that ECV was statistically different between HHD and HCM, while the remaining three concluded that it was not statistically significant. Given that the small number of included studies results in each study playing a high effect weight in the overall result, removing any of the positive studies would further reduce the already modest result.

### 4.1 Diffuse fibrosis

Increased T1 mapping, used to quantify the longitudinal relaxation time of tissues (49), is mainly due to edema and expanded interstitial space (50). Because both HHD and HCM pathologically exhibit myocyte hypertrophy and interstitial fibrosis (51, 52), without significant myocardial edema, T1 mapping may predominantly reflect interstitial fibrosis levels, with HCM showing more fibrosis than HHD. However, as T1 mapping combines signals from both myocytes and extracellular volume (50), differences in hypertrophic patterns and degrees could influence T1 mapping. ECV, derived from pre- and post-contrast T1 mapping of myocardium and blood (50), might be a more reliable indicator of diffuse interstitial fibrosis, minimizing the influence of cardiomyocytes (53–55). Hence, lower ECV in HHD compared to HCM more confidently indicates increased interstitial fibrosis in HCM than T1 mapping alone. However, the accuracy and stability of both are contingent on various factors like field strengths, sequences, vendors, post-processing software and methods, contributing to heterogeneity. Postprocessing with multilayer averaging has less heterogeneity and obtains more stable results than single-layer, probably due to the former better reflecting the heart as a whole. The majority of Asia group studies likely used exactly identical machines and protocols due to being from the same institution, contributing to their notably lower heterogeneity. Grouping T1 mapping by EF difference significantly lowered heterogeneity, indicating that it may reflect severity difference between HHD and HCM (56, 57) is a source of heterogeneity and influences T1 mapping variability and stability.

### 4.2 Myocardial deformation

Myocardial strain, a dimensionless index measuring myocardial deformation (58), is superior to EF in describing myocardial deformation and identifying abnormalities of function (59, 60). Myocardial strains (GRS, GCS and GLS) are primarily computed using CMR feature tracking (CMR-FT) with semi-automatically outlining the myocardial contour on cine sequences (61). Myocardial strain is influenced by intracellular, extracellular (62), and molecular myocardial components (63). Despite known differences in these components between HHD and HCM (1, 2), our study found no statistical significance in global strains across all three directions. It's possible that global strains lost subtle information, like variations in specific segments, cardiac layers and strain rate, leading to the non-appearance of differences. For instance, in HCM, early subendocardial layer involvement (64) and asymmetric hypertrophy resulting in uneven segmental effects are not precisely captured by global strain. GLS was significantly less heterogeneous in both subgroups of vendor, highlighting it as a significant source of heterogeneity. Additionally, semi-automatic human contouring (65) of the myocardium, image quality, post-processing software and different regions lead to variability in the data, which affects the final pooled effect and heterogeneity.

### 4.3 Structure and function

Cardiomyocyte hypertrophy and interstitial hyperplasia, stemming from different etiologies, lead to increased ventricular mass and wall thickness (66), affecting diastolic and systolic function (67). HHD shows LV hypertrophy as a response to increased afterload, initially maintaining normal wall stress and function (concentric hypertrophy) (68) but eventually causing LV dilatation and reduced function (eccentric hypertrophy) (57). And this hypertrophy is usually moderate, especially in the early stages of the disease. HCM, due to genetic mutations (69), typically characterized by asymmetric LV thickening without chamber dilatation (70), exhibits normal or supernormal systolic function but impaired diastolic function (71). LVMI primarily indicates the extent of cardiac hypertrophy, commonly assessing heart remodeling in HHD, whereas Maximal LVWT gauges the ventricular wall's peak thickness, often used in diagnosing HCM and its subtypes. ESVI and EDVI adjust for individual differences and more accurately reflect systolic and diastolic functions. In our study, HCM exhibited higher LVMI and Maximal LVWT, but lower ESVI and EDVI than HHD, indicating greater myocardial hypertrophy and more pronounced diastolic function impairment but better systolic function in HCM. However, the substantial overlap in cardiac hypertrophy and diastolic-systolic alterations between HHD and HCM implies no absolute value can definitively differentiate them. To the best of our knowledge, high heterogeneity likely stems from variations in disease duration, complications, medication, ethnic backgrounds of patients, image quality and operator variability in myocardial contouring.

Apart from the previously mentioned factors, about 50% of the included studies reported late gadolinium enhancement (LGE); however, its presence or absence was not a continuous variable, so it could not be meta-analyzed. Nonetheless, we discovered that the majority of the research reported the presence of LGE in 25%–35% of HHD and more than 80% of HCM, indicating that HCM is more likely to be associated with myocardial fibrosis or scarring.

The absolute value of Hedges'g represents the discrimination power, with high effect for Maximal LVWT and ESVI, medium for EDVI, and low for T1mapping, ECV and LVMI. Unfortunately, the current study's limitations prevent us from establishing a strong specific value to quasi-differentiate them. However, by combining data from multiple studies, this meta-analysis enhances statistical power and reliability, providing a comprehensive overview that makes the conclusions more robust and dependable than those of individual studies. Our study demonstrates that HCM patients exhibit significantly higher levels of diffuse fibrosis, as indicated by elevated T1 mapping and ECV. High T1 mapping and ECV values serve as reliable biomarkers for HCM, enhancing early diagnostic accuracy and helping clinicians differentiate between HCM and HHD more effectively, thus reducing the risk of misdiagnosis. Early detection of higher diffuse fibrosis levels in patients with HCM facilitates timely antifibrotic interventions that have the potential to slow disease progression, reduce complication rates, and improve patient prognosis. Although strain parameters did not show significant differences, highlighting the importance of integrating multiple CMR parameters in clinical evaluations remains crucial. Additionally, structural and functional indices aid in disease differentiation and severity assessment, guiding risk evaluation and management strategies to enhance long-term outcomes. Overall, these CMR parameters improve diagnostic precision and treatment personalization, leading to better patient care and outcomes.

## 5 Limitations

Concerning limitations, (1) this review predominantly included observational and retrospective studies, whose inherent limitations may affect result interpretation. Observational studies are prone to confounding factors and retrospective studies may suffer from selection bias and missing data, impacting the accuracy and consistency of the results; (2) the reliability of the results may be weakened by a high degree of heterogeneity, and the results should be interpreted with caution. Despite extensive subgroup and regression analyses, the source of heterogeneity for some indicators remains unclear; (3) the publication bias in GLS and ESVI analyses, instability of ECV in sensitivity analysis, and limited number of studies in certain subgroups call for confirmation of these findings in further studies; (4) the clinical heterogeneity and slight differences in diagnostic criteria of HHD and HCM, and our lack of focus on specific subgroups like obstructive and nonobstructive HCM, may affect results; (5) many studies lacked sufficient data for exploring potential covariates such as complications and disease duration; (6) while meta-analysis is a useful tool, our limited statistical power means the results should be interpreted cautiously.

## 6 Future directions

Although certain CMR parameters exhibit statistical differences between HHD and HCM, their limited discriminatory capacity highlights the need for integrated and enhanced approaches like algorithms combining multiple parameters (16) or deep learning (20). Considering the heterogeneity of myocardial hypertrophy, better stability and differentiation can be achieved by measuring T1 mapping and ECV in more myocardial layers. And comprehensive analysis using bull’s-eye plots of segmental strain (18, 29), strain rate (18, 35), multilayer strain (subendocardial, mid-myocardial, subepicardial) (16) and left atrial strain (15) may reveal differences obscured by global strains. Future research should also validate discriminatory efficacy of indicators like steep left ventricle to aortic root angle (72), diastolic septal perforator flow velocities (73). Additionally, three-dimensional heart modelling based on MR, proven to be highly reproducible and more informative (74–76), may differentiate myocardial morphology between HHD and HCM (77). Moreover, cardiac diffusion tensor imaging (DTI) (78, 79), may effectively distinguishes between HHD and HCM by analyzing myocardial fiber bundle features (like alignment, orientation, and integrity), especially given the disordered cardiomyocyte arrangement in HCM (80). Investigating these diseases across subgroups and stages will improve diagnostic accuracy and understanding of disease progression. In conclusion, the evolving CMR technology and increasing patient population underscores the importance and potential for future research in this field.

## 7 Conclusion

Our meta-analysis reveals that multiple CMR parameters play an important role in distinguishing HHD from HCM, underscores the pathophysiological distinctions underlying these parameter differences, and highlights the importance of considering specific multiple factors in clinical interpretation. The findings also underscore the importance of standardized CMR protocols due to the impact of certain relevant variables on outcomes. These insights are essential for enhancing diagnostic precision and optimizing treatment strategies for HHD and HCM patients, while further research is needed to advance the diagnosis and understanding of these conditions.

## Data Availability

Publicly available datasets were analyzed in this study. This data can be found here: this was a meta-analysis and all included articles were carefully cited.
