# Supplementary material for: Cardiac magnetic resonance imaging for discrimination of hypertensive heart disease and hypertrophic cardiomyopathy: a systematic review and meta-analysis
Source: Front Cardiovasc Med. 2024 Aug 2;11:1421013. doi: 10.3389/fcvm.2024.1421013 (PMC11327824; doi:10.3389/fcvm.2024.1421013)
Supplement: Supplementary file 1 [file Datasheet1.pdf]

Supplementary Material

Supplementary Methods

A. Decomposition of means and Standard Deviation from two subgroups

Tool: CombineMeanSD (statstodo.com): Cochrane's Formula

1.Combined n = n1 + n2

2.Combined mean = (n1*m1 + n2*m2) / (n1 + n2)

3.Combined Standard Deviation = sqrt(((n1-1) *s1*s1 + (n2-1) *s2*s2 + n1 * n2 / (n1 + n2) * (m1*m1 + m2*m2 - 2 * m1 * m2)) / (n1 + n2 -1));

B. Estimating the sample mean and standard deviation from sample minimum, median and maximum if the data are not significantly skewed away from normality

Tool: Mean Variance Estimation (hkbu.edu.hk) : Scenario 1

Estimated mean of the sample from Luo et al. (2018)[1].

Estimated standard deviation of the sample from Wan et al. (2014)[2].

C. Convert SEM (Standard error of mean) to SD (Standard error of mean)

SD=SEM* sqrt(N)

Supplementary Table S1. Search strategy and number of results.

Databases		PubMed (195), Embase (2564), Web of Science (132), Cochrane Library (8)
Date of search		September 2023
Search strategy	HHD	"HHD" OR "Hypertensive Heart Disease" OR "hypertension-induced left ventricular hypertrophy" OR "H-LVH" OR "Hypertensive cardiomyopathy" OR "Hypertension-related cardiac hypertrophy"
	HCM	"HCM" OR "Hypertrophic Cardiomyopathy" OR "Genetic hypertrophic cardiomyopathy"
	MRI	CMR" OR "Cardiac Magnetic Resonance" OR "Cardiac MR*" OR "T1mapping" OR "Extracellular volume" OR "ECV" OR "Global longitudinal strain" OR "Global circumferential strain" OR

		"Global radial strain" OR "Myocardial strain" OR "LV mass" OR "Maximal LV wall thickness" OR "Cardiac function" OR "Left ventricular function"
	(HHD related keywords) AND (HCM related keywords) AND (MRI related keywords)	

Supplementary Fig S1. Publication bias of CMR parameters.

A. T1mapping

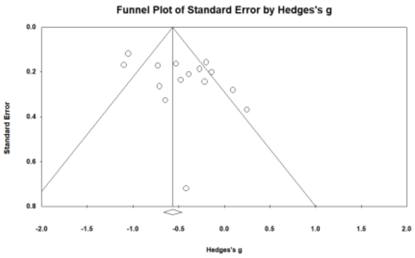

B. ECV

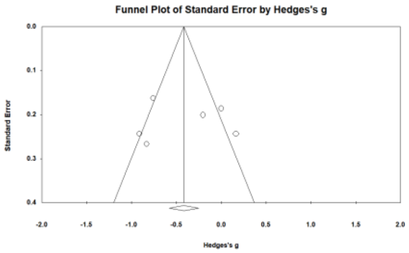

C. GRS

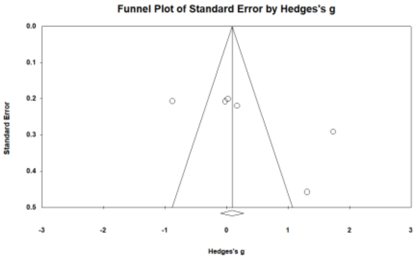

D. GCS

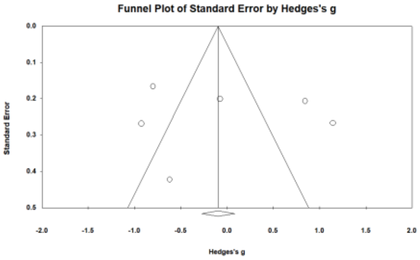

E. GLS

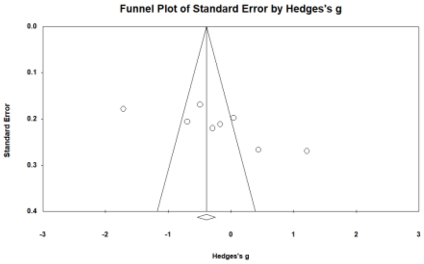

F. LVMI

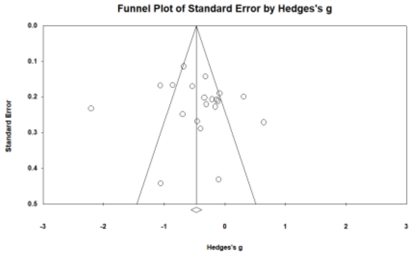

G. Maximal LVWT

H. ESVI

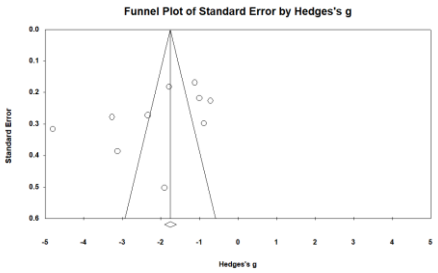

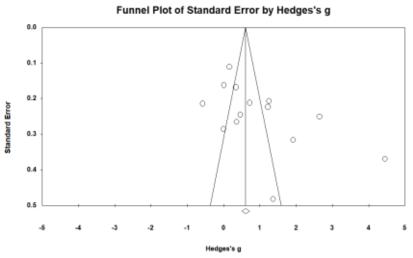

I. EDVI

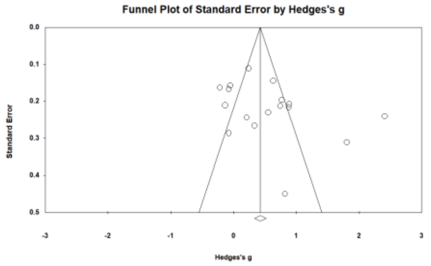

Supplementary Table S2. Quality assessment checklist.

Study	Q1	Q2	Q3	Q4	Q5	Q6	Q7	Q8
Kong et al. (2023)	Y/Y	Y/Y	Y/Y	Y/Y	Y/Y	Y/Y	Y/Y	Y/Y
Hao et al. (2023)	Y/Y	Y/Y	Y/Y	Y/Y	Y/Y	Y/Y	Y/Y	Y/Y
Wang et al. (2022)	Y/Y	Y/Y	Y/Y	Y/Y	Y/Y	Y/Y	Y/Y	Y/Y
Yao et al. (2023)	Y/Y	Y/Y	Y/Y	Y/Y	Y/Y	Y/Y	Y/Y	Y/Y
Liu et al. (2023)	Y/Y	Y/Y	Y/Y	Y/Y	Y/Y	Y/Y	Y/Y	Y/Y
Lavall et al. (2023)	Y/Y	Y/Y	Y/Y	Y/Y	N/N	N/N	Y/Y	Y/Y
Liu et al. (2022)	Y/Y	Y/Y	Y/Y	Y/Y	N/N	N/N	Y/Y	Y/Y
Liang et al. (2022)	Y/Y	Y/Y	Y/Y	Y/Y	N/N	N/N	Y/Y	Y/Y
Hirschberg et al. (2022)	Y/Y	Y/Y	Y/Y	Y/Y	N/N	N/N	Y/Y	Y/N
Giusca et al. (2021)	Y/Y	Y/Y	Y/N	Y/Y	N/N	Y/N	Y/Y	Y/Y
Zhan et al. (2021)	Y/Y	Y/Y	Y/Y	Y/Y	N/N	N/N	Y/Y	Y/Y
Shi et al. (2021)	Y/Y	Y/Y	Y/Y	Y/Y	N/N	N/N	Y/Y	Y/Y
Arcari et al. (2020)	Y/Y	Y/Y	Y/N	Y/Y	N/N	N/N	Y/Y	Y/Y
Satriano et al. (2020)	Y/Y	Y/Y	Y/Y	Y/Y	N/N	N/N	Y/Y	Y/Y
Neisius et al. (2019)	Y/Y	Y/Y	Y/Y	Y/Y	N/N	N/N	Y/Y	Y/Y
Jiang et al. (2018)	Y/Y	Y/Y	Y/Y	Y/Y	Y/Y	N/N	Y/Y	Y/Y
Chacko et al. (2018)	Y/Y	Y/Y	Y/Y	Y/Y	Y/Y	N/N	Y/Y	Y/Y

Arenja et al. (2017)	Y/Y	Y/Y	Y/N	Y/Y	Y/Y	N/N	Y/Y	Y/Y
Wu et al. (2017)	Y/Y	Y/Y	Y/Y	Y/Y	Y/Y	N/N	Y/Y	Y/Y
Rodrigues et al. (2017)	Y/Y	Y/Y	Y/Y	Y/Y	Y/Y	N/N	Y/Y	Y/Y
Hinojar et al. (2015)	Y/Y	Y/Y	Y/Y	Y/Y	N/N	N/N	Y/Y	Y/Y
Takeda et al. (2013)	Y/Y	Y/Y	Y/N	Y/Y	N/N	N/N	Y/Y	Y/Y
Sipola et al. (2011)	Y/Y	Y/Y	Y/Y	Y/Y	N/N	N/N	Y/Y	Y/Y
Puntmann et al. (2010)	Y/Y	Y/Y	Y/Y	Y/Y	N/N	N/N	Y/Y	Y/Y
Piella et al. (2010)	Y/Y	Y/Y	Y/Y	Y/Y	N/N	N/N	Y/Y	N/N
Petersen et al. (2005)	Y/Y	Y/Y	Y/Y	Y/Y	N/N	N/N	Y/Y	Y/N

NOTE: This table is given by two raters (Q.Z. and Z.C.), Q1. Were the criteria for inclusion in the sample clearly defined? Q2. Were the study subjects and the setting described in detail? Q3. Was the exposure measured in a valid and reliable way? Q4. Were objective, standard criteria used for measurement of the condition? Q5. Were confounding factors identified? Q6. Were strategies to deal with confounding factors stated? Q7. Were the outcomes measured in a valid and reliable way? Q8. Was appropriate statistical analysis used? Y Yes, N No

Supplementary Table S3. Results of the subgroup analyses.

Parameter	Subgroup	Variable	No. of studies	Hedges'g			Z-value	P-value	Heterogeneity	
				g	Lower g	Upper g			I2 (%)	p
T1mapping	B0 field	1.5T	2	-0.712	-1.040	-0.384	-4.253	<0.001*	<0.001	0.675
		3T	12	-0.433	-0.693	-0.174	-3.272	0.001*	79.173	<0.001*
	software	notCvi42	3	-0.512	-0.811	-0.213	-3.360	0.001*	34.503	0.217
		Cvi42	11	-0.393	-0.628	-0.158	-3.279	0.001*	66.268	0.001*
	vendor	Siemens	4	-0.345	-0.988	0.299	-1.050	0.294	87.571	<0.001*
		Philips	10	-0.485	-0.714	-0.255	-4.134	<0.001*	64.466	0.003*
	region	Asia	7	-0.283	-0.440	-0.127	-3.545	<0.001*	2.803	0.404
		Western	8	-0.642	-0.927	-0.357	-4.417	<0.001*	71.799	0.001*
	Slice	=1	6	-0.693	-1.044	-0.341	-3.861	<0.001*	73.017	0.002*
		>1	9	-0.346	-0.514	-0.177	-4.030	<0.001*	33.644	0.149
	△EF	≤5	6	-0.833	-1.058	-0.608	-7.252	<0.001*	50.182	0.074
		>5	8	-0.217	-0.367	-0.068	-2.850	0.004*	<0.001	0.663
ECV	software	notCvi42	2	-0.373	-1.430	0.683	-0.693	0.488	89.775	0.002*
		Cvi42	4	-0.436	-0.842	-0.030	-2.104	0.035*	76.810	0.005*
	region	Asia	4	-0.460	-0.905	-0.016	-2.030	0.042*	76.163	0.006*
		Western	2	-0.314	-1.218	0.590	-0.681	0.496	89.923	0.002*
GRS	B0 field	1.5T	3	1.050	-0.045	2.145	1.879	0.060	89.893	<0.001*
		3T	3	-0.290	-0.862	0.282	-0.994	0.320	83.540	0.002*
	software	notCvi42[a]	2	0.070	-0.225	0.366	0.465	0.642	<0.001	0.524
		Cvi42	3	0.280	-1.059	1.619	0.409	0.682	96.263	<0.001*
	vendor	GE	2	-0.177	-0.641	0.287	-0.747	0.455	<0.001	0.433
		Philips	4	1.613	1.132	2.094	6.575	<0.001*	80.639	0.001*
	region	Asia	4	0.198	-0.721	1.117	0.422	0.673	94.396	<0.001*
		Western	2	0.671	-0.437	1.779	1.187	0.235	80.214	0.025*
GCS	B0 field	1.5T	2	0.294	-1.433	2.021	0.334	0.739	91.973	<0.001*

Parameter	Subgroup	Variable	No. of studies	Hedges'g			Z-value	P-value	Heterogeneity	
				g	Lower g	Upper g			I2 (%)	p
		3T	3	-0.040	-0.988	0.909	-0.082	0.935	92.916	<0.001*
	vendor	GE	2	0.294	-1.433	2.021	0.334	0.739	91.973	<0.001*
		Philips	3	-0.040	-0.988	0.909	-0.082	0.935	92.916	<0.001*
	region	Asia	4	0.250	-0.593	1.094	0.582	0.561	92.714	<0.001*
		Western	2	-0.775	-1.078	-0.472	-5.012	<0.001*	<0.001	0.689
GLS	B0 field	1.5T	4	0.198	-0.540	0.936	0.525	0.599	90.966	<0.001*
		3T	3	-0.270	-0.704	0.163	-1.224	0.221	71.318	0.031*
	software	notCvi42	2	-1.010	-2.403	0.383	-1.422	0.155	96.022	<0.001*
		Cvi42	6	0.035	-0.463	0.534	0.138	0.890	88.133	<0.001*
	vendor	Philips	4	-0.361	-0.667	-0.056	-2.321	0.020*	59.862	0.058
		Siemens	2	0.114	-0.480	0.708	0.377	0.706	68.600	0.074
	region	Asia	3	0.175	-0.825	1.174	0.343	0.732	93.676	<0.001*
		Western	5	-0.458	-1.156	0.239	-1.288	0.198	93.415	<0.001*
	△EF	≤5	4	-0.152	-1.350	1.047	-0.248	0.804	96.976	<0.001*
		>5	4	-0.275	-0.587	0.037	-1.729	0.084	57.057	0.072
LVMI	B0 field	1.5T	8	-0.276	-0.549	-0.003	-1.982	0.047*	61.108	0.012*
		3T	10	-0.526	-0.897	-0.155	-2.777	0.005*	90.827	<0.001*
	software	notCvi42	8	-0.579	-1.060	-0.097	-2.357	0.018*	88.390	<0.001*
		Cvi42	9	-0.269	-0.602	0.064	-1.582	0.114	85.557	<0.001*
	vendor	Philips	13	-0.475	-0.792	-0.158	-2.940	0.003*	87.464	<0.001*
		Siemens	5	-0.166	-0.610	0.279	-0.731	0.465	83.019	<0.001*
	region	Asia	7	-0.412	-0.966	0.142	-1.457	0.145	92.320	<0.001*
		Western	13	-0.457	-0.681	-0.232	-3.990	<0.001*	74.260	<0.001*
	△EF	≤5	11	-0.380	-0.633	-0.127	-2.946	0.003*	79.763	<0.001*
		>5	8	-0.581	-1.111	-0.051	-2.147	0.032*	90.861	<0.001*
Maximal LVWT	B0 field	1.5T	6	-2.286	-3.582	-0.990	-3.458	0.001*	96.293	<0.001*
		3T	4	-1.773	-2.616	-0.931	-4.127	<0.001*	94.282	<0.001*
	software	notCvi42	6	-2.155	-3.371	-0.939	-3.472	0.001*	97.005	<0.001*
		Cvi42	3	-1.942	-2.828	-1.057	-4.299	<0.001*	91.911	<0.001*
	vendor	Philips	7	-2.234	-3.186	-1.282	-4.599	<0.001*	96.705	<0.001*
		Siemens	2	-1.610	-3.033	-0.186	-2.217	0.027*	92.261	<0.001*
	region	Asia	3	-2.003	-3.146	-0.861	-3.436	0.001*	95.136	<0.001*
		Western	7	-2.111	-3.151	-1.072	-3.981	<0.001*	96.095	<0.001*
	△EF	≤5	5	-2.166	-3.497	-0.836	-3.192	0.001*	97.127	<0.001*
		>5	4	-1.908	-2.969	-0.848	-3.527	<0.001*	94.097	<0.001*
ESVI	B0 field	1.5T	5	0.647	0.119	1.175	2.402	0.016*	84.110	<0.001*
		3T	7	1.347	0.381	2.313	2.733	0.006*	97.453	<0.001*
	software	notCvi42	6	0.996	0.218	1.774	2.510	0.012*	93.401	<0.001*
		Cvi42	6	1.208	0.073	2.344	2.085	0.037*	97.221	<0.001*
	vendor	Philips	9	1.574	0.860	2.287	4.321	<0.001*	94.881	<0.001*
		Siemens	4	-0.015	-0.404	0.374	-0.076	0.940	72.421	0.012*
	region	Asia	5	2.177	1.113	3.242	4.009	<0.001*	94.796	<0.001*
		Western	9	0.346	0.010	0.682	2.017	0.044*	84.755	<0.001*
	△EF	≤5	10	0.522	0.136	0.908	2.653	0.008*	89.885	<0.001*
		>5	4	2.227	0.523	3.932	2.561	0.010*	96.733	<0.001*
EDVI	B0 field	1.5T	6	0.528	0.032	1.024	2.087	0.037*	85.442	<0.001*
		3T	9	0.638	0.222	1.054	3.003	0.003*	92.025	<0.001*
	software	notCvi42	7	0.671	0.007	1.335	1.980	0.048*	92.758	<0.001*
		Cvi42	8	0.475	0.078	0.873	2.343	0.019*	88.494	<0.001*

Parameter	Subgroup	Variable	No. of studies	Hedges'g			Z-value	P-value	Heterogeneity	
				g	Lower g	Upper g			I2 (%)	p
	vendor	Philips	11	0.802	0.389	1.216	3.802	<0.001*	91.092	<0.001*
		Siemens	5	0.196	-0.026	0.419	1.728	0.084	34.854	0.189
	region	Asia	6	1.066	0.558	1.573	4.114	<0.001*	88.273	<0.001*
		Western	11	0.267	0.003	0.530	1.984	0.047*	80.419	<0.001*
	△EF	≤5	11	0.355	0.063	0.647	2.380	0.017*	84.922	<0.001*
		>5	5	0.994	0.280	1.708	2.728	0.006*	92.114	<0.001*

NOTE: Some of the subgroups not performed were due to the small number of studies within subgroups (n<2), B0 field: Magnetic field strength of the MR used, software: Software used for post-processing, vendor: Vendors of MR, region: Region in which the Institute is located, △EF: Absolute value of the difference between the mean ejection fraction of HHD and HCM; notCvi42: Software other than cvi42, e.g., ViewForum; Abbreviations: ECV Extracellular Volume, GRS Global radial strain, GCS Global circumferential strain, GLS Global longitudinal strain, LVMI Left ventricular mass index, LVWT Left ventricular wall thickness, ESVI End-systolic-volume index, EDVI End-diastolic-volume index, [a] only ViewForum, * significant at p < 0.05

Supplementary Table S4. Results of the regression analysis.

Parameter	Moderator	No. of studies	Regression coefficient	Standard error	Lower limit	Upper limit	T-Value	P-Value
T1mapping	Age (y)	15	-0.014	0.022	-0.062	0.034	-0.640	0.531
	Sex (male %)	14	0.016	0.015	-0.017	0.050	1.080	0.302
	EF	14	0.015	0.022	-0.033	0.063	0.680	0.511
LVMI	Age (y)	20	-0.022	0.026	-0.077	0.033	-0.860	0.403
	Sex (male %)	20	0.004	0.014	-0.025	0.033	0.280	0.786
	EF (%)	19	-0.012	0.017	-0.049	0.024	-0.730	0.477
Maximal LVWT	Age (y)	10	-0.058	0.082	-0.247	0.132	-0.700	0.503
	Sex (male %)	10	-0.060	0.042	-0.157	0.038	-1.41	0.195
	EF (%)	9	0.031	0.094	-0.192	0.254	0.330	0.752
ESVI	Age (y)	14	-0.041	0.073	-0.199	0.117	-0.560	0.584
	Sex (male %)	14	0.020	0.037	-0.060	0.101	0.550	0.592
	EF (%)	14	-0.033	0.036	-0.112	0.045	-0.930	0.371
EDVI	Age (y)	17	-0.009	0.034	-0.081	0.622	-0.270	0.788
	Sex (male %)	17	0.015	0.020	-0.028	0.057	0.740	0.469
	EF (%)	16	0.002	0.022	-0.044	0.048	0.090	0.928

NOTE: Parameters not performed could not be analyzed in regression analyses due to small numbers (n<10), The covariates were all calculated using the weighted mean of HHD and HCM; Abbreviations: LVMI Left ventricular mass index, LVWT Left ventricular wall thickness, ESVI End-systolic-volume index, EDVI End-diastolic-volume index

Supplementary Fig S2. Sensitivity analysis using the one-study-removed method.

A. T1mapping

Sensitivity analysis of T1mapping

Study name	Point	Standard error	Variance	Lower limit	Upper limit	Z-Value	p-Value
Kong et al. (2023)	-0.474	0.115	0.013	-0.699	-0.248	-4.112	0.000
Wang et al. (2023)	-0.494	0.112	0.013	-0.713	-0.274	-4.402	0.000
Lavall et al. (2023)	-0.487	0.113	0.013	-0.707	-0.266	-4.324	0.000
Liang et al. (2022)	-0.467	0.115	0.013	-0.692	-0.242	-4.064	0.000
Hirschberg et al. (2022) H1.5	-0.470	0.110	0.012	-0.685	-0.254	-4.268	0.000
Hirschberg et al. (2022) H3	-0.458	0.113	0.013	-0.680	-0.237	-4.057	0.000
Hirschberg et al. (2022) M	-0.505	0.107	0.011	-0.715	-0.295	-4.711	0.000
Giusca et al. (2021)	-0.461	0.118	0.014	-0.693	-0.229	-3.898	0.000
Shi et al. (2021)	-0.496	0.111	0.012	-0.714	-0.278	-4.466	0.000
Arcari et al. (2020)	-0.420	0.097	0.009	-0.609	-0.230	-4.339	0.000
Neisius et al. (2019)	-0.445	0.117	0.014	-0.674	-0.216	-3.810	0.000
Jiang et al. (2018)	-0.485	0.114	0.013	-0.708	-0.261	-4.246	0.000
Wu et al. (2017)	-0.452	0.114	0.013	-0.675	-0.229	-3.966	0.000
Hinojar et al. (2015)	-0.418	0.105	0.011	-0.625	-0.212	-3.967	0.000
Zhan et al. (2021)	-0.506	0.108	0.012	-0.718	-0.295	-4.699	0.000
	-0.469	0.108	0.012	-0.681	-0.258	-4.352	0.000

(Statistics with study removed)

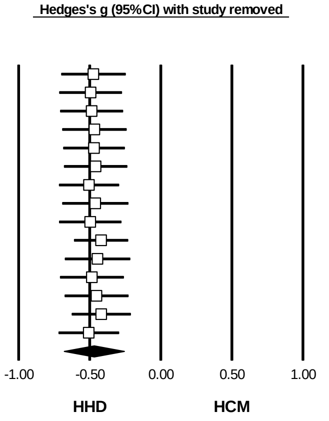

B. ECV

Sensitivity analysis of ECV

Study name	Point	Standard error	Variance	Lower limit	Upper limit	Z-Value	p-Value
Lavall et al. (2023)	-0.524	0.187	0.035	-0.891	-0.158	-2.802	0.005
Liang et al. (2022)	-0.325	0.197	0.039	-0.710	0.061	-1.651	0.099
Shi et al. (2021)	-0.463	0.222	0.049	-0.899	-0.027	-2.081	0.037
Jiang et al. (2018)	-0.507	0.199	0.039	-0.897	-0.118	-2.554	0.011
Wu et al. (2017)	-0.344	0.204	0.042	-0.744	0.056	-1.687	0.092
Hinojar et al. (2015)	-0.340	0.206	0.042	-0.744	0.064	-1.649	0.099
	-0.417	0.185	0.034	-0.779	-0.055	-2.258	0.024

(Statistics with study removed)

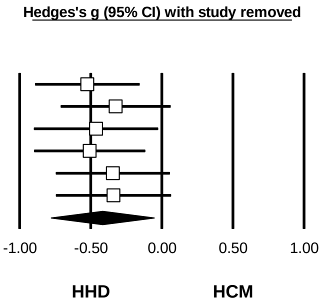

C. GRS

Sensitivity analysis of GRS

Study name	Point	Standard error	Variance	Lower limit	Upper limit	Z-Value	p-Value
Kong et al. (2023)	0.435	0.438	0.192	-0.423	1.293	0.993	0.320
Hao et al. (2023)	0.037	0.267	0.071	-0.487	0.560	0.137	0.891
Liu et al. (2022)	0.591	0.327	0.107	-0.050	1.231	1.808	0.071
Shi et al. (2021)	0.427	0.442	0.196	-0.440	1.294	0.965	0.335
Puntmann et al. (2010)	0.394	0.433	0.187	-0.454	1.242	0.910	0.363
Piella et al. (2010)	0.188	0.363	0.132	-0.524	0.900	0.517	0.605
	0.346	0.346	0.120	-0.331	1.024	1.001	0.317

(Statistics with study removed)

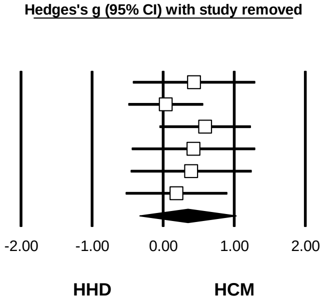

D. GCS

Sensitivity analysis of GCS

Study name	Point	Standard error	Variance	Lower limit	Upper limit	Z-Value	p-Value
Hao et al. (2023)	-0.299	0.351	0.123	-0.987	0.389	-0.852	0.394
Liu et al. (2022)	-0.250	0.366	0.134	-0.967	0.467	-0.683	0.495
Giusca et al. (2021)	0.097	0.385	0.148	-0.657	0.852	0.253	0.800
Shi et al. (2021)	-0.061	0.459	0.211	-0.962	0.839	-0.134	0.894
Wu et al. (2017)	0.110	0.398	0.158	-0.670	0.890	0.277	0.782
Piella et al. (2010)	0.034	0.402	0.161	-0.753	0.821	0.085	0.932
	-0.061	0.360	0.130	-0.767	0.644	-0.171	0.865

Hedges's g (95% CI) with study removed

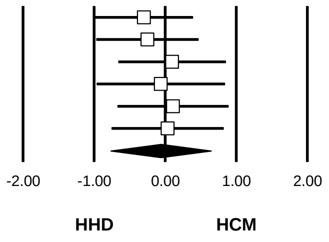

E. GLS

Sensitivity analysis of GLS

Study name	Point	Standard error	Variance	Lower limit	Upper limit	Z-Value	p-Value
Hao et al. (2023)	-0.422	0.263	0.069	-0.938	0.093	-1.605	0.108
Liu et al. (2022)	-0.259	0.331	0.110	-0.908	0.389	-0.783	0.433
Giusca et al. (2021)	-0.015	0.217	0.047	-0.441	0.411	-0.069	0.945
Shi et al. (2021)	-0.153	0.334	0.112	-0.807	0.502	-0.457	0.647
Satriano et al. (2020)	-0.228	0.335	0.112	-0.884	0.427	-0.682	0.495
Neisius et al. (2019)	-0.180	0.348	0.121	-0.863	0.503	-0.517	0.605
Rodrigues et al. (2017)	-0.314	0.311	0.097	-0.923	0.296	-1.008	0.313
Puntmann et al. (2010)	-0.210	0.334	0.112	-0.865	0.445	-0.629	0.529
	-0.223	0.292	0.085	-0.794	0.349	-0.763	0.445

Hedges's g (95% CI) with study removed

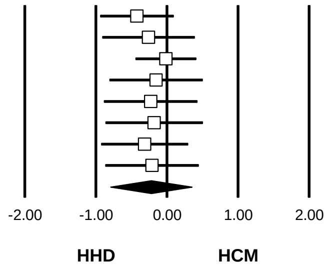

F. LVMI

Sensitivity analysis of LVMI

Study name	Point	Standard error	Variance	Lower limit	Upper limit	Z-Value	p-Value
Kong et al. (2023)	-0.454	0.125	0.016	-0.698	-0.209	-3.636	0.000
Yao et al. (2023)	-0.457	0.125	0.016	-0.701	-0.213	-3.670	0.000
Lavall et al. (2023)	-0.424	0.125	0.016	-0.668	-0.180	-3.402	0.001
Liu et al. (2022)	-0.479	0.118	0.014	-0.711	-0.248	-4.053	0.000
Giusca et al. (2021)	-0.413	0.125	0.016	-0.657	-0.168	-3.305	0.001
Shi et al. (2021)	-0.443	0.126	0.016	-0.690	-0.196	-3.513	0.000
Arcari et al. (2020)	-0.423	0.130	0.017	-0.678	-0.167	-3.243	0.001
Satriano et al. (2020)	-0.455	0.125	0.016	-0.699	-0.211	-3.655	0.000
Neisius et al. (2019)	-0.432	0.128	0.016	-0.681	-0.182	-3.385	0.001
Jiang et al. (2018)	-0.348	0.094	0.009	-0.532	-0.164	-3.708	0.000
Chacko et al. (2018)	-0.437	0.125	0.016	-0.681	-0.192	-3.501	0.000
Arenja et al. (2017)	-0.450	0.125	0.016	-0.695	-0.204	-3.588	0.000
Rodrigues et al. (2017)	-0.490	0.116	0.013	-0.718	-0.263	-4.221	0.000
Hinojar et al. (2015)	-0.401	0.121	0.015	-0.638	-0.164	-3.312	0.001
Takeda et al. (2013)	-0.449	0.123	0.015	-0.689	-0.209	-3.667	0.000
Sipola et al. (2011)	-0.452	0.125	0.016	-0.696	-0.208	-3.628	0.000
Puntmann et al. (2010)	-0.444	0.125	0.016	-0.690	-0.199	-3.543	0.000
Liu et al. (2023)	-0.444	0.129	0.017	-0.697	-0.192	-3.453	0.001
Piella et al. (2010)	-0.416	0.122	0.015	-0.655	-0.177	-3.409	0.001
Petersen et al. (2005)	-0.439	0.124	0.015	-0.683	-0.195	-3.530	0.000
	-0.437	0.120	0.014	-0.672	-0.203	-3.654	0.000

Hedges's g (95% CI) with study removed

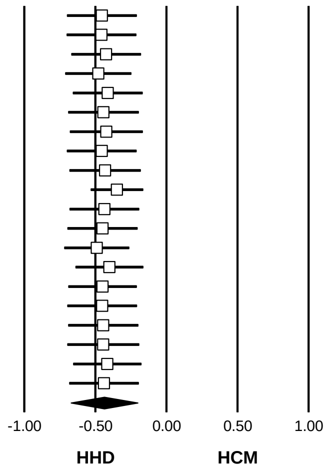

G. Maximal LVWT

Sensitivity analysis of Maximal LVWT

Statistics with study removed

Study name	Point	Standard error	Variance	Lower limit	Upper limit	Z-Value	p-Value
Kong et al. (2023)	-2.200	0.412	0.170	-3.007	-1.393	-5.344	0.000
Wang et al. (2022)	-2.112	0.440	0.193	-2.974	-1.251	-4.804	0.000
Neisius et al. (2019)	-1.764	0.290	0.084	-2.333	-1.195	-6.075	0.000
Jiang et al. (2018)	-1.941	0.380	0.144	-2.685	-1.197	-5.111	0.000
Chacko et al. (2018)	-1.964	0.391	0.153	-2.730	-1.199	-5.029	0.000
Hinojar et al. (2015)	-2.189	0.425	0.180	-3.021	-1.356	-5.153	0.000
Sipola et al. (2011)	-2.048	0.410	0.168	-2.852	-1.244	-4.992	0.000
Puntmann et al. (2010)	-2.232	0.398	0.158	-3.012	-1.452	-5.607	0.000
Piella et al. (2010)	-2.093	0.399	0.159	-2.874	-1.312	-5.250	0.000
Petersen et al. (2005)	-2.209	0.402	0.162	-2.997	-1.421	-5.493	0.000
	-2.076	0.374	0.140	-2.808	-1.343	-5.551	0.000

Hedges's g (95% CI) with study removed

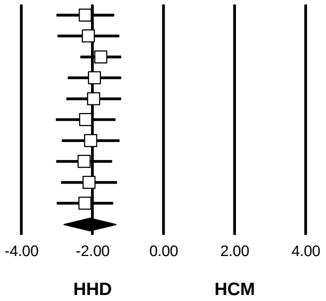

H. ESVI

Sensitivity analysis of ESVI

Statistics with study removed

Study name	Point	Standard error	Variance	Lower limit	Upper limit	Z-Value	p-Value
Kong et al. (2023)	0.977	0.286	0.082	0.417	1.536	3.420	0.001
Yao et al. (2023)	0.974	0.286	0.082	0.414	1.535	3.406	0.001
Lavall et al. (2023)	1.036	0.288	0.083	0.473	1.600	3.604	0.000
Liu et al. (2022)	0.737	0.221	0.049	0.305	1.170	3.342	0.001
Giusca et al. (2021)	1.075	0.291	0.085	0.505	1.645	3.696	0.000
Arcari et al. (2020)	1.066	0.305	0.093	0.468	1.663	3.497	0.000
Satriano et al. (2020)	1.116	0.274	0.075	0.580	1.652	4.079	0.000
Neisius et al. (2019)	1.049	0.296	0.088	0.468	1.629	3.540	0.000
Jiang et al. (2018)	0.857	0.250	0.062	0.368	1.347	3.432	0.001
Chacko et al. (2018)	0.924	0.275	0.076	0.385	1.463	3.358	0.001
Arenja et al. (2017)	1.017	0.291	0.085	0.447	1.587	3.498	0.000
Rodrigues et al. (2017)	1.044	0.286	0.082	0.483	1.604	3.649	0.000
Takeda et al. (2013)	0.969	0.279	0.078	0.423	1.515	3.478	0.001
Petersen et al. (2005)	1.070	0.283	0.080	0.515	1.624	3.780	0.000
	0.993	0.269	0.072	0.467	1.519	3.699	0.000

Hedges's g (95% CI) with study removed

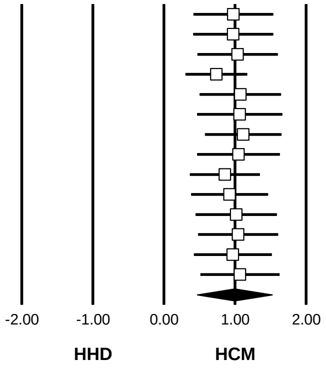

H. EDVI

Sensitivity analysis of EDVI

Study name	Point	Standard error	Variance	Lower limit	Upper limit	Z-Value	p-Value
	Statistics with study removed						
Kong et al. (2023)	0.533	0.157	0.025	0.226	0.840	3.400	0.001
Yao et al. (2023)	0.540	0.158	0.025	0.230	0.850	3.411	0.001
Lavall et al. (2023)	0.575	0.158	0.025	0.266	0.884	3.645	0.000
Liu et al. (2022)	0.532	0.157	0.025	0.225	0.839	3.394	0.001
Giusca et al. (2021)	0.605	0.153	0.023	0.304	0.905	3.946	0.000
Arcari et al. (2020)	0.577	0.167	0.028	0.249	0.905	3.450	0.001
Satriano et al. (2020)	0.597	0.156	0.024	0.292	0.902	3.839	0.000
Neisius et al. (2019)	0.596	0.157	0.025	0.288	0.903	3.799	0.000
Jiang et al. (2018)	0.427	0.117	0.014	0.198	0.657	3.649	0.000
Chacko et al. (2018)	0.482	0.146	0.021	0.196	0.769	3.300	0.001
Arenja et al. (2017)	0.542	0.158	0.025	0.232	0.852	3.428	0.001
Rodrigues et al. (2017)	0.567	0.157	0.025	0.258	0.875	3.600	0.000
Hinojar et al. (2015)	0.595	0.157	0.025	0.286	0.903	3.778	0.000
Takeda et al. (2013)	0.542	0.154	0.024	0.239	0.844	3.508	0.000
Sipola et al. (2011)	0.554	0.158	0.025	0.244	0.865	3.498	0.000
Liu et al. (2023)	0.550	0.163	0.026	0.230	0.869	3.376	0.001
Petersen et al. (2005)	0.590	0.155	0.024	0.285	0.894	3.792	0.000
	0.553	0.150	0.023	0.259	0.847	3.685	0.000

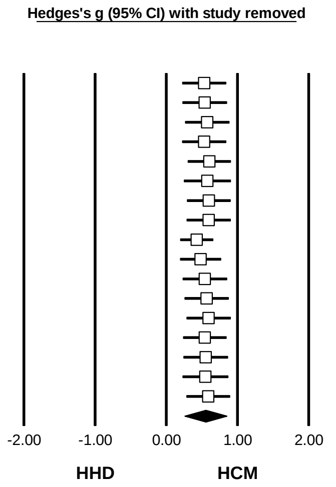

References

[1] D. Luo, X. Wan, J. Liu, and T. Tong, 'Optimally estimating the sample mean from the sample size, median, mid-range, and/or mid-quartile range', *Stat Methods Med Res*, vol. 27, no. 6, pp. 1785–1805, Jun. 2018, doi: 10.1177/0962280216669183.

[2] X. Wan, W. Wang, J. Liu, and T. Tong, 'Estimating the sample mean and standard deviation from the sample size, median, range and/or interquartile range', *BMC Med Res Methodol*, vol. 14, no. 1, p. 135, Dec. 2014, doi: 10.1186/1471-2288-14-135.
